# Therapeutic Effect and Mechanism of* Oxytropis falcata* Gel on Deep Second-Degree Burn in Rats

**DOI:** 10.1155/2017/3729547

**Published:** 2017-11-13

**Authors:** Xiao-Feng Lin, Kai-Jie Chen, He-Kun Shi, Le Yu, Jin-Shan Chen, Yan Fei

**Affiliations:** ^1^Department of Pharmacy, The 175th Hospital of PLA, Affiliated Southeast Hospital of Xiamen University, Fujian, Zhangzhou 363000, China; ^2^Department of Pathology, The 175th Hospital of PLA, Affiliated Southeast Hospital of Xiamen University, Fujian, Zhangzhou 363000, China

## Abstract

*Oxytropis falcata *has long been used to treat inflammation, sores, and bleeding in Tibet. However, the burn remedy and underlying molecular mechanisms are not well understood. This study is aimed at assessing the effect of* Oxytropis falcate *gel (OFG) on deep second-degree burn rats and exploring its mechanism. Wistar rats with second-degree burn were treated with OFG and silver sulfadiazine. Immunohistochemical detections for EGF and VEGF were performed, and ELISA detections for EGF, VEGF, p38, and IL-1*β* in serum were determined. Rats treated with OFG (25, 50 g/kg) consisted of the major rhamnocitrin-3-O-*β*-neohesperidoside significantly accelerated incrustation (*P* < 0.001) and decrustation (*P* < 0.001). According to HE staining, edema and infiltration of inflammatory cells decrease apparently with good hyperplasia and incrustation in administration groups (7 d). The expressions of EGF and CD34 in OFG (25, 50 g/kg) treatment increased obviously from immunohistochemical assessment (7 d). Serum EGF expression reached 321.27 ± 7.20 ng/mL by OFG treatment, while p38 (*P* < 0.05) and IL-1*β* (*P* < 0.05) levels were significantly lower than the model and vehicle groups from day 1 to day 7. OFG possesses potential wound healing activities. The mechanism may be related to the increasing of biosynthesis and the releasing of EGF and CD34 and the decreasing p38 and IL-1*β* levels.

## 1. Introduction

Burning can cause all sorts of tissue damage depending on burn severity. Burns can be classified as first-, second-, and third-degree burns according to the involvement of skin and deeper tissues. Burns are so much different from other injuries that a separate medical superspeciality has been designated to treat them [1]. Nowadays, prescription drugs such as sulfadiazine silver and mafenide, which are effective in relieving symptoms and promoting healing, are widely used in burn. These sulfonamides show a satisfactory antibacterial effect. However, the wound contraction heals slowly because multiple biological pathways need to be activated and synchronized to respond [2]. The wound repair process can be accelerated by recombinant human fibroblast growth factor (rhFGF), basic fibroblast growth factor (bFGF), and collagenase, but the antibacterial effect is limited. Recently there was a new trend in characterizing active constituents from the Chinese ethnodrugs [3].

*Oxytropis falcata* Bunge (Leguminosae), known as “Er-Da-Xia” in Tibetan medicines, is a wild growing plant mainly distributed in Qinghai-Tibet Plateau at an altitude of 2700–4300 m in China. This plant has been used as folk remedies to treat inflammation, sores, and bleeding for thousands of years [4–6]. Moreover, some traditional patented prescriptions containing this herb have also been launched into the market. However, to our knowledge, the effect on burn treatment of* Oxytropis falcate *has never been reported in academic research. Therefore, in this study, we extracted the components of* Oxytropis falcata* Bunge and observed effects of OFG on rats with deep second-degree burn to explore the mechanism using pharmacological experiment and molecular biotechnology.

## 2. Materials and Methods

### 2.1. Drugs and Chemicals

*Oxytropis falcata* herb was obtained from The Huangheyuan medicinal materials company in Gansu Province. The plant was identified and authenticated by Professor Zhigang Ma at Lanzhou University (Lanzhou, China). Borneol and water-soluble chitosan (molecular weight 200 kDa, 90% deacetylation) were provided by Qingdao Shunbo Biotechnology Institute Company Limited. Take 1% silver sulfadiazine cream as positive control. Rats EGF, VEGF, p38, and IL-1*β* ELISA kits were obtained from American R&D Company. Rabbit anti-EGF and anti-VEGF-*β* were acquired from Boster Biological Company Limited (China). Rabbit anti-CD34 and immunohistochemisty strengthen kit (Envision TM plus) were obtained from Fuzhou Maixin Biotechnology Company Limited (China).

### 2.2. Animals

All male and female Wistar rats weighing 200–250 g were purchased from Shanghai SLAC Laboratory Animals Co., Ltd. (China). The 98 rats were randomly selected and divided into 7 groups: model group, vehicle group, administration groups (low-dose, middle-dose, and high-dose group), positive group, and normal group. They were housed in clear plastic cages with solid floors and hard wood chip bedding and placed in a temperature- and humidity-controlled environment. The experiment was conducted in the central lab of Affiliated Dongnan Hospital of Xiamen University in accordance with laboratory animal standards in China (GB14925-95) and the University Guidelines of the Ethics Committee for Animal Care and Use in Research. The experiment was performed according to the 3R principle of animals use and blinded treatment.

### 2.3. Prescription Preparation and Administration

The herb (200 g) was cut into small pieces and extracted with purified water (400 mL, 1.5 h reflux, 100°C, ×2). The water solution was concentrated under a reduced pressure after filtration in a Buchner funnel, enriching the total flavones by macroporous resin. The extract content of rhamnocitrin-3-O-*β*-neohesperidoside was 6.46% with a diode-array detector at 350 nm by RP-HPLC [7].

The extract (60 g, crude drug 15 g/g) was resuspended in distilled water with continuous stirring. Then chitosan (60 g) was added to the stirring mixture till it became swollen completely. Next, borneol (10 g, dissolved into 20 mL glycerol) was added. Subsequently, sodium benzoate (1.2 g) was cast over the gel. Finally, volume was made with distilled water and was stirred continuously till a uniform high-dose gel weighing 1000 g formed. The gel (pH 6.0–7.0) had translucent appearance, good spreadability, uniform particle size distribution, and moderate viscosity (5.73 Pa·s). And the extracts in the middle-dose and low-dose gels were 50% and 25% of high-dose, respectively. The vehicle control gel was prepared without the extract. Model group were deep second-degree burn rats without administration, and normal group were rats without modeling and without administration. Vehicle (10 g/kg), low-dose, middle-dose, high-dose (12.5, 25 and 50 g/kg), and positive (silver sulfadiazine, 10 g/kg) group were painted for administration twice a day for 28 days to monitor adverse drug reactions and other accident situations. The doses and dose interval were used according to the preliminary laboratory experiment.

### 2.4. Deep Second-Degree Burn Modeling

The model was built according to the scalded rat model from Fu and Wang [8]. An area (5 cm × 5 cm) was shaved on the rat back. The rats were anesthetized by the vapor of ethyl alcohol. Then cardboard with circular hole (4 cm in diameter) was prepared for burned areas. The shaved animal skin was burned for 12 s with 3 drops of ethanol. Then the fire was put off as soon as possible with a wet cloth. Moreover, the depth was confirmed by observing the pathological change with hematoxylin and eosin (HE) staining. A deep partial-thickness burn injury was observed in rats. Epidermis disappeared. Epidermis, dermis, and subcutaneous tissue were partially damaged. There were obvious subcutaneous tissue edema and infiltration of inflammatory cells. The wounds were observed daily until a complete wound healing enclosure occurred.

### 2.5. Immunohistochemistry

The wound tissue sections were fixed in 4% formaldehyde. Then they were penetrated with paraffin and paraffin-embedded according to conventional methods. HE staining was conducted on days 7, 14, and 21 after burn injury. For this study 4 *μ*m thick sections were retained on poly-L-lysine coated slides and baked for 2 h at 68°C. Paraffin sections were dewaxed and rinsed with tap water. Antigen retrieval was achieved by boiling for 4 min in citrate buffer (pH 6.0). After natural cooling, endogenous peroxidase activity was blocked by using 3% hydrogen peroxide for 10 min. Next sections were washed three times with Phosphate Buffered Saline (PBS, pH 7.6). After this stage, sections were incubated with primary antibody for 1 h at 37°C and then washed three times with PBS. Then sections were incubated with secondary antibody for 15 min at room temperature and washed three times with PBS. Finally, the color was developed by 5 min incubation with diaminobenzidine solution after contrasting with hematoxylin. In order to evaluate the epithelization and angiogenesis process in rat endothelium epitope, we used anti-EGF, anti-VEGF, and anti-CD34 antibody.

### 2.6. ELISA

The rats were anesthetized by the vapor of ethyl alcohol after burn injury on days 1, 4, and 7, to take 2 mL blood at the inner canthus with glass capillary tube. Blood was allowed to coagulate for 15 min at room temperature. Then 200 *μ*L supernatant was collected after the blood was centrifugated at 1000*g* for 15 min. TGF-*β*, p38, and IL-1*β* were detected according to the instructions.

### 2.7. Statistical Analysis

Statistical analysis was performed using SPSS software version 17. All experimental parameters were expressed as the mean ± the standard error mean (SEM). Statistical comparisons were made using one-way analysis of variance (ANOVA) followed by LSD's post hoc test. *P* values less than 0.05 were considered to be statistically significant.

## 3. Results

### 3.1. Evaluation of Tissue Repair

**Figure 1:**
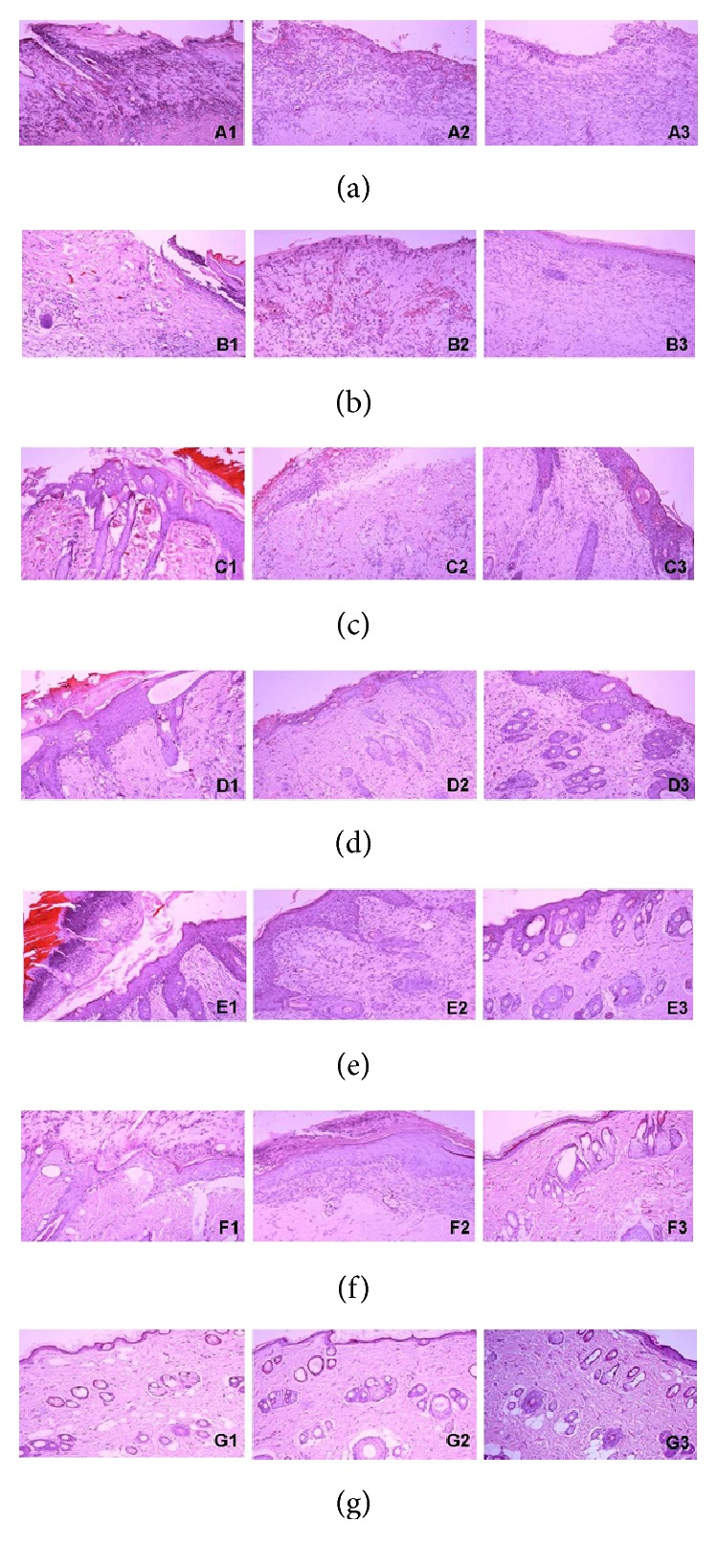
Therapeutic effect of OFBGC on rats with II° burn (HE staining, ×100). The measurements were carried out on day 7, day 14, and day 21. Red areas showed incrustation and purple particles were inflammatory cells. (a) model group, (b) vehicle control, (c) low-dose group, (d) middle-dose group, (e) high-dose group, (f) positive control, and (g) normal control. 1: 7 d, 2: 14 d, and 3: 21 d.

The gels could serve as wound dressings to prepare an optimum wound bed without secretion. In OFG-treated groups, oedema and infiltration of inflammatory cells apparently decreased in burnt areas with good hyperplasia and incrustation (7 d). On day 14, new hair follicle and sebaceous glands were observed with almost complete epithelization and decrustation, in contrast with the incomplete epithelization in model and vehicle groups. On day 21, the administration rats showed a complete healing process contrasting the poor situation in model groups (Figure 1).

Incrustation is the solid covering or layer that is formed from necrotic tissue. Decrustation is the scab being removed from skin surface with complete wound closure [9]. The administration and vehicle rats showed significantly less time needed for incrustation (*P* < 0.001) and decrustation (*P* < 0.001) as compared with model rats. Moreover, significantly less time was needed for incrustation in vehicle, middle-dose, and high-dose groups than positive group (*P* < 0.001), and the incrustation and decrustation time in high-dose group was shorter than positive control (*P* < 0.001) (Table 1).

### 3.2. p38, IL-1*β*, EGF, and VEGF Production in Serum

The productions of p38 and IL-1*β* were augmented in model rats as compared with normal rats (*P* < 0.05) on days 1, 4, and 7. However, the levels of p38 in low-dose (7 d), middle-dose (1, 4, and 7 d), and high-dose groups (1, 4, and 7 d) were significantly lower than that in model group (*P* < 0.05). The level of IL-1*β* in low-dose (1, 4 d), middle-dose (1, 4, and 7 d), and high-dose groups (1, 4, and 7 d) were significantly lower than that in model group (*P* < 0.05). Rats with OFG expressed significantly lower levels of p38 and IL-1*β* compared with those without OFG (vehicle group), as shown in Table 2. There were significant increases (*P* < 0.05) in EGF in low-dose (314.39 ± 10.18 ng/mL) and middle-dose rats (321.27 ± 7.20 ng/mL) as compared with model rats. But the production of VEGF in OFG-treated rats was found to be a little different from other groups. Moreover, the OFG-treated rats also showed significant increases (*P* < 0.05) in EGF and VEGF as compared with the vehicle group (Table 3).

### 3.3. EGF, VEGF, and CD34 Production in Tissue

**Figure 2:**
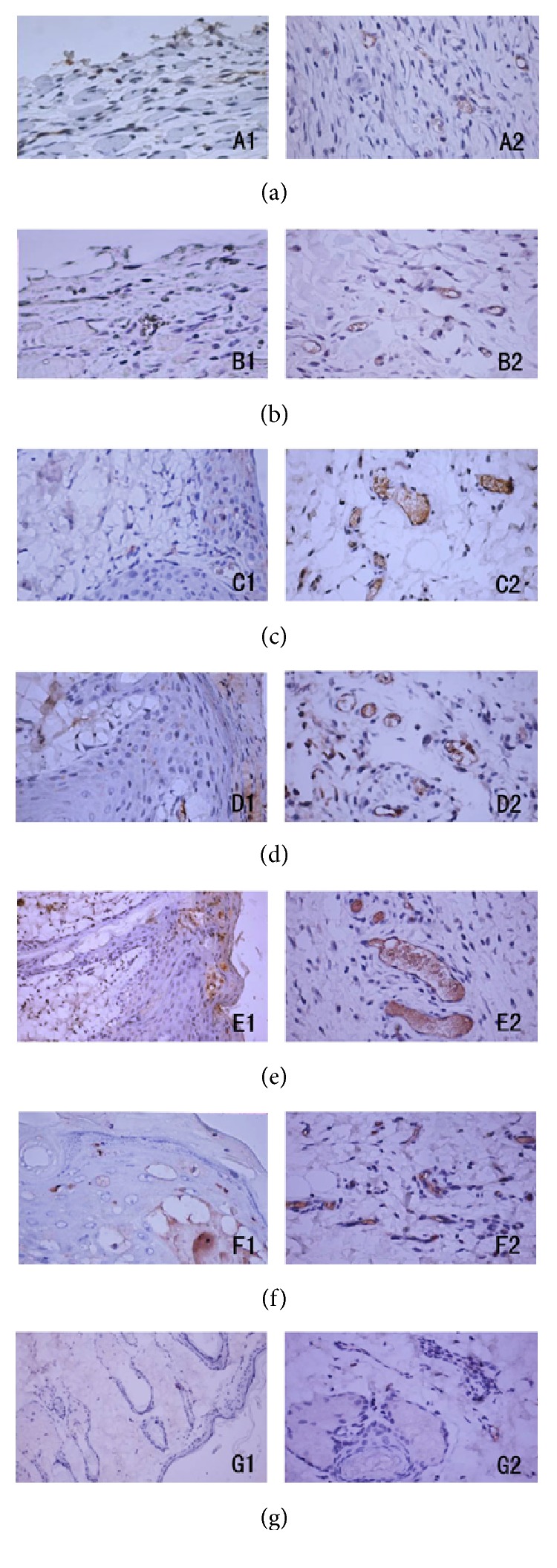
EGF and VEGF expression of immunohistochemical (×400) at day 7. The fuscescent ones, respectively, represented EGF and VEGF protein at different doses. 1: EGF and 2: VEGF; (a) model group, (b) vehicle control, (c) low-dose group, (d) middle-dose group, (e) high-dose group, (f) positive control, and (g) normal control.

On day 7, the model, vehicle, and normal control groups showed no obvious increase in EGF and VEGF in burnt tissues compared with the normal group. But there were significant expressions of EGF in low-dose and middle-dose rats. The administration rats also showed obvious increases in VEGF (Figure 2).

**Figure 3:**
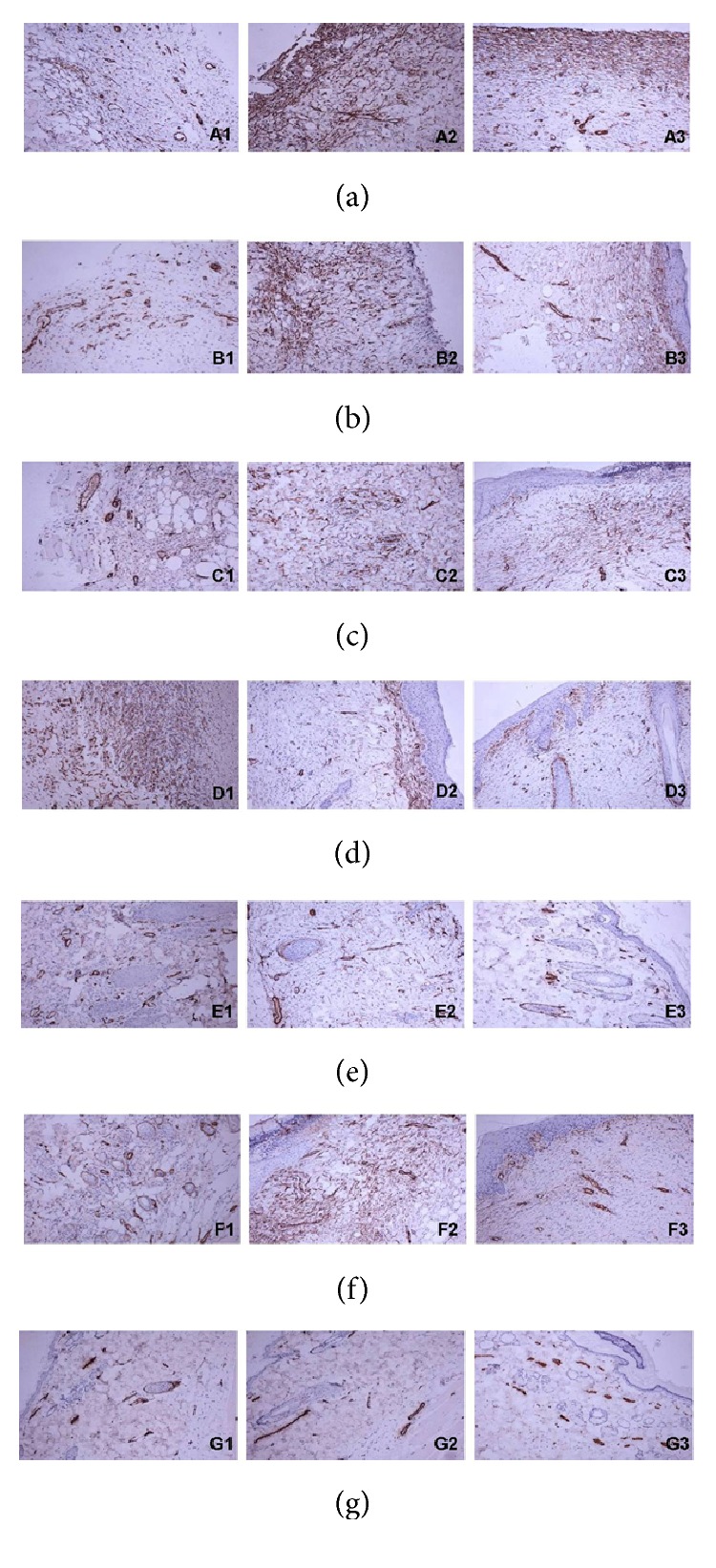
CD34 expression of immunohistochemical (×400). The staining CD34 protein (fuscescent ones) could represent blood vessel. The capillary was new generated in the reparative process and the coarse vessels were existing. (a) Model group, (b) vehicle control, (c) low-dose group, (d) middle-dose group, (e) high-dose group, (f) positive control, and (g) normal control. 1: 7 d, 2: 14 d, and 3: 21 d.

Normal rats were found to be of no obvious expression of CD34 because no capillary formed. There were increases in CD34 in OFG-treated rats on day 7, especially in middle-dose group, and the expression in middle-dose rats was observed in subepidermic layer on day 14 and day 21. However, the model group showed a delayed increase in CD34 from day 7 to day 21 (Figure 3).

## 4. Discussion

In our study, the major constituent rhamnocitrin-3-O-*β*-neohesperidoside, a flavonoid, in our extract was 6.46%. So rhamnocitrin-3-O-*β*-neohesperidoside and other flavones present in the extract are responsible for the effective epithelialization with good hyperplasia and incrustation in burn-injured rats. Some compounds in the gel would presumably have antibacterial properties because the gels could apparently decrease oedema and infiltration of inflammatory cells without secretion. These results are closely related to the reports. The total flavonoids from* Oxytropis falcate* possessed anti-inflammatory, antioxidant, and ultraviolet protective effects on the destructed skin [10] and antibacterial activities against nine Gram-positive and Gram-negative bacteria, especially* Staphylococcus aureus* [11].

Extract of* Oxytropis falcate* treatment induced moderate liver damage and mild renal damage with maximum oral gavage dose for 15 days [12]. However, our previous study suggested that OFG had good absorption and no obvious skin toxicity [13]. Externally applied agents of* Oxytropis falcate* may be a relatively safe drug and take less risk than oral and intravenous preparations. Hence, as a potential preparation, OFG needs to be further tried on small, clinical wounds and large animals wounds before being applied to humans in the future.

Although the wound healing in vehicle-treated rats was not significant when compared with OFG-treated rats, it did show improved results in comparison with no treatment rats. The probable reason was that chitosan could prevent the loss of body fluid, prevent exudate buildup, protect the wounds from external contamination, have sufficient bactericidal activity to inhibit infection, and prepare an optimum wound bed for tissue repairing [14]. OFG accelerated wound healing, comparing with vehicle and model groups where the healing was delayed and almost spontaneous. The potential mechanisms are closely related to abnormal expressions of some cytokines in inflammatory reaction and regulation of growth factors, which are in line with findings of previous workers [15].

EGF binding to its receptor (EGFR) triggers rapid human skin fibroblast and keratinocyte locomotion. Additionally, this process plays a critical role in cell migration and human cutaneous wounds [16], not in the closure of an open wound [17]. In fact, burn wounds in OFG-treated rats were healing well by way of incrustation and decrustation, rather than gradual closure. The migrating mechanism may depend on EGFR-mediated expression of aquaporin-3 in a time- and dose-dependent manner [18]. We observed EGF expression in low-dose and middle-dose group, while no significant upregulation occurred in high-dose and normal group in serum at the same time. There are many possible reasons why the high dose was not observed to increasing EGF as we expected. Firstly, the upregulation of EGF is likely to be influenced by the presence of some active proteins, as it is also regulated in the matched unburned skin of the rats. Secondly, there were increasing EGF in burnt skin in rats, rather than in serum. It means that maybe the releasing behavior of EGF in the serum possibly occurred earlier than in burn wound. The other reason may be that EGF is a specific marker for wound repair in tissue rather than in blood. However, it suggests that it recommended further studies.

Similar studies on VEGF were carried out. OFG treatment can accelerate VEGF expression in a dose-dependent manner in healing tissue, but weak expression in the low-dose group was observed in serum on day 7. VEGF could promote angiogenesis by promoting the formation of endothelial cells and inducing newly formed blood vessels in wound healing [19]. However, it is different from our study. Maybe VEGF is just a secondary bystander in OFG treatment. But, in our research CD34 may contribute directly to wound healing in the event of angiogenesis, which are in line with previous findings. A good angiogenesis was observed in OFG treatment following the significant increase CD34 expression. Strings of CD34 endothelial precursor cells were observed at the edges on day 3 after burn injury. The density of newly formed blood vessels on the surface unit within granulation tissue grew from 3 to 12 days [20].

Different from positive control, OFG-treated groups took less healing time for wound repair and showed more effective epithelialization by enhancing expression of EGF and in II° burn rats (Table 1, Figures 2 and 3). And they showed significant anti-inflammatory action (Table 2). Immediate or delayed topical application for p38 inhibitor remains potent in reducing full-thickness burning inflammatory signaling [21]. Similar findings were observed in our study where the expressions of p38 and IL-1*β* were significantly decreased in OFG-treated rats on days 1, 4, and 7 after burn injury. Hence, p38 and its downstream factor IL-1*β* may be the representative targets for inflammatory reaction in burn wound healing for OFG treatment.

## 5. Conclusions

OFG can not only remarkably intensify incrustation and decrustation processes but also relieve the inflammatory reaction with deep second-degree burn. Based on these findings, the potential mechanisms are possibly related to the increasing synthesis and releasing of EGF and CD34 in wound healing, decreasing expression of p38 and IL-1*β*. Therefore, OFG is recommended to be a potential compound for burn remedy. In order to provide new evidence in clinic based treatment strategies for patients with OFG, these results should be sufficiently confirmed by further experiments.

## Figures and Tables

**Table 1:** Healing time of wound in II° burn rats.

Treatment	Incrustation time (days)	Decrustation time (days)
Model	8.29 ± 0.57^*###*^	22.25 ± 0.56^*###*^
Vehicle	3.16 ± 0.42^*∗∗∗*,###^	16.91 ± 0.46^*∗∗∗*,###^
Low-dose	2.31 ± 0.32^*∗∗∗*,###^	13.77 ± 0.39^*∗∗∗*^
Middle-dose	2.16 ± 0.34^*∗∗∗*,###^	12.18 ± 0.58^*∗∗∗*,###^
High-dose	1.88 ± 0.35^*∗∗∗*,###^	9.38 ± 0.51^*∗∗∗*,###^
Positive	4.14 ± 0.45^*∗∗∗*^	13.96 ± 0.60^*∗∗∗*^

*Note*. ^*∗∗∗*^*P* < 0.001 versus model group. ^###^*P* < 0.001 versus positive control.

**Table 2:** Expression of p38 and IL-1*β* in II° burns rat serum.

Treatment	p38			IL-1		
	1 d	4 d	7 d	1 d	4 d	7 d
Model	79.89 ± 2.95^#^	79.98 ± 4.37^#^	80.92 ± 3.57^#^	26.31 ± 1.66^#^	27.68 ± 2.08^#^	26.84 ± 1.66^#^
Vehicle	79.25 ± 3.58^#^	76.42 ± 4.17	77.29 ± 4.13	25.23 ± 1.99	26.34 ± 1.43	25.83 ± 1.58
Low-dose	76.21 ± 4.41	76.06 ± 4.72	75.70 ± 4.20^*∗*^	24.70 ± 1.96^*∗*^	25.67 ± 1.65^*∗*^	25.40 ± 1.19
Middle-dose	75.32 ± 2.88^*∗*^	72.04 ± 4.95^*∗*^	72.34 ± 4.23^*∗*^	23.77 ± 1.66^*∗*^	25.60 ± 1.98^*∗*^	24.82 ± 1.53^*∗*^
High-dose	72.40 ± 3.86^*∗*^	72.38 ± 4.81^*∗*^	72.29 ± 4.22^*∗*^	23.67 ± 1.87^*∗*^	24.70 ± 1.25^*∗*^	24.46 ± 1.28^*∗*^
Positive	74.32 ± 5.12^*∗*^	73.16 ± 3.84^*∗*^	73.26 ± 3.26^*∗*^	23.36 ± 1.50^*∗*^	23.35 ± 1.11^*∗*^	23.50 ± 1.29^*∗*^
Normal	74.40 ± 4.91^*∗*^	74.39 ± 4.62^*∗*^	74.31 ± 4.31^*∗*^	24.13 ± 1.22^*∗*^	25.12 ± 1.49^*∗*^	24.85 ± 1.31^*∗*^

*Note*. ^*∗*^*P* < 0.05 versus model group. ^#^*P* < 0.05 versus positive control.

**Table 3:** Expression of EGF and VEGF in II° burns rat serum.

Treatment	EGF	VEGF
Model	305.81 ± 15.52	100.29 ± 7.42
Vehicle	310.60 ± 13.84	99.14 ± 7.07
Low-dose	314.39 ± 10.18^#^	110.07 ± 1.35^#^
Middle-dose	321.27 ± 7.20^*∗*,#^	104.40 ± 3.53
High-dose	307.98 ± 10.03	101.98 ± 4.38
Positive	318.17 ± 8.07^#^	102.89 ± 4.19
Normal	299.49 ± 16.28	101.47 ± 3.42

*Note*. ^*∗*^*P* < 0.05 versus model group. ^#^*P* < 0.05 versus normal control.
